# Author Correction: Superconducting Diamond on Silicon Nitride for Device Applications

**DOI:** 10.1038/s41598-019-46256-y

**Published:** 2019-07-24

**Authors:** Henry A. Bland, Evan L. H. Thomas, Georgina M. Klemencic, Soumen Mandal, David J. Morgan, Andreas Papageorgiou, Tyrone G. Jones, Oliver A. Williams

**Affiliations:** 1 School of Physics and Astronomy, Cardiff University, Queen’s Building, The Parade, Cardiff, CF24 3AA UK; 2 Cardiff Catalysis Institute, School of Chemistry, Cardiff University, Park Place, Cardiff, CF10 3AT Wales UK; 3 QMC Instruments Ltd., School of Physics and Astronomy, Cardiff University, Queen’s Building, The Parade, Cardiff, CF243AA UK

Correction to: *Scientific Reports* 10.1038/s41598-019-39707-z, published online 27 February 2019

In Figure 4, the ‘RCA-1 cleaned’ and ‘Solvent cleaned’ plots are labelled incorrectly. The correct Figure 4 appears below as Figure 1.

**Figure 1:**
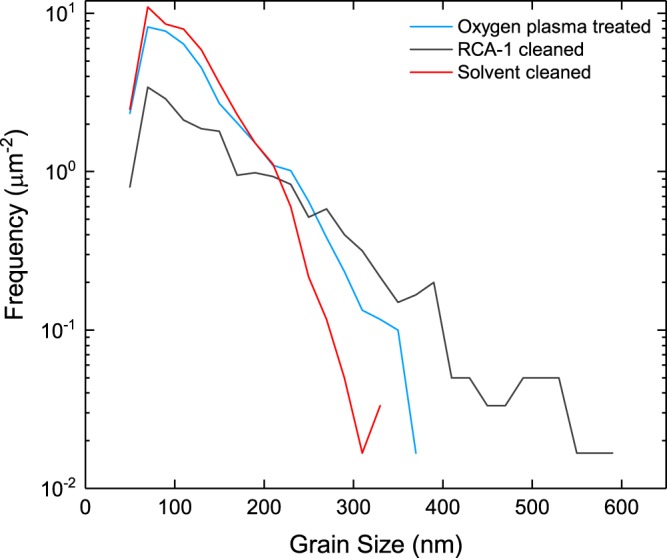
A plot displaying the grain size distribution for each diamond film.

